# Health impact assessment of the UK soft drinks industry levy: a comparative risk assessment modelling study

**DOI:** 10.1016/S2468-2667(16)30037-8

**Published:** 2016-12-16

**Authors:** Adam D M Briggs, Oliver T Mytton, Ariane Kehlbacher, Richard Tiffin, Ahmed Elhussein, Mike Rayner, Susan A Jebb, Tony Blakely, Peter Scarborough

**Affiliations:** a British Heart Foundation Centre on Population Approaches for Non-Communicable Disease Prevention, Nuffield Department of Population Health, University of Oxford, Headington, Oxford, UK; b UK Clinical Research Collaboration Centre for Diet and Activity Research and Medical Research Council Epidemiology Unit, University of Cambridge School of Clinical Medicine, Cambridge, UK; c Agri-Food Economics and Social Sciences Division, School of Agriculture, Policy and Development, University of Reading, Reading, UK; d Oxford University Medical School, Medical Sciences Division, University of Oxford, John Radcliffe Hospital, Oxford, UK; e Nuffield Department of Primary Care Health Sciences, University of Oxford, Radcliffe Observatory Quarter, Oxford, UK; f Health Inequalities Research Programme, Department of Public Health, University of Otago, Wellington, New Zealand

## Abstract

**Background:**

In March, 2016, the UK Government proposed a tiered levy on sugar-sweetened beverages (SSBs; high tax for drinks with >8 g of sugar per 100 mL, moderate tax for 5–8 g, and no tax for <5 g). We estimate the effect of possible industry responses to the levy on obesity, diabetes, and dental caries.

**Methods:**

We modelled three possible industry responses: reformulation to reduce sugar concentration, an increase of product price, and a change of the market share of high-sugar, mid-sugar, and low-sugar drinks. For each response, we defined a better-case and worse-case health scenario. We developed a comparative risk assessment model to estimate the UK health impact of each scenario on prevalence of obesity and incidence of dental caries and type 2 diabetes. The model combined data for sales and consumption of SSBs, disease incidence and prevalence, price elasticity estimates, and estimates of the association between SSB consumption and disease outcomes. We drew the disease association parameters from a meta-analysis of experimental studies (SSBs and weight change), a meta-analysis of prospective cohort studies (type 2 diabetes), and a prospective cohort study (dental caries).

**Findings:**

The best modelled scenario for health is SSB reformulation, resulting in a reduction of 144 383 (95% uncertainty interval 5102–306 743; 0·9%) of 15 470 813 adults and children with obesity in the UK, 19 094 (6920–32 678; incidence reduction of 31·1 per 100 000 person-years) fewer incident cases of type 2 diabetes per year, and 269 375 (82 211–470 928; incidence reduction of 4·4 per 1000 person-years) fewer decayed, missing, or filled teeth annually. An increase in the price of SSBs in the better-case scenario would result in 81 594 (3588–182 669; 0·5%) fewer adults and children with obesity, 10 861 (3899–18 964; 17·7) fewer incident cases of diabetes per year, and 149 378 (45 231–262 013; 2·4) fewer decayed, missing, or filled teeth annually. Changes to market share to increase the proportion of low-sugar drinks sold in the better-case scenario would result in 91 042 (4289–204 903; 0·6%) fewer adults and children with diabetes, 1528 (4414–21 785; 19·7) fewer incident cases of diabetes per year, and 172 718 (47 919–294 499; 2·8) fewer decayed, missing, or filled teeth annually. The greatest benefit for obesity and oral health would be among individuals aged younger than 18 years, with people aged older than 65 years having the largest absolute decreases in diabetes incidence.

**Interpretation:**

The health impact of the soft drinks levy is dependent on its implementation by industry. Uncertainty exists as to how industry will react and about estimation of health outcomes. Health gains could be maximised by substantial product reformulation, with additional benefits possible if the levy is passed on to purchasers through raising of the price of high-sugar and mid-sugar drinks and activities to increase the market share of low-sugar products.

**Funding:**

None.

## Introduction

In 2015, the UK Scientific Advisory Committee on Nutrition published a report^1^ on the evidence for the association between consumption of carbohydrates and health. The report clarified the role of sugar for development of dental caries and identified sugar-sweetened beverages (SSBs) as a specific risk factor for weight gain and type 2 diabetes, recommending that SSB consumption should be minimised. Both Public Health England^2^ and the UK House of Parliament's Health Committee^3^ subsequently advised a tax on SSBs and, in March, 2016, the budget statement^4^ included proposals for a soft drinks industry levy.

Taxes on SSBs have been previously introduced in Mexico, France, Hungary, and elsewhere;^5^ however, the UK would be the first to introduce a three-tiered levy. The levy is presented as an incentive for the industry to reformulate existing products to remove sugar, reduce portion sizes, and promote new or existing low-sugar alternatives. The levy is due to be introduced in 2018, subject to parliament passing the legislation in 2017, with revenue hypothecated for an increase of physical activity and breakfast clubs in schools.^4^

Although the UK Government has expressed a desire that the levy is not passed on to purchasers through price rises, this request cannot be mandated and the industry response is unknown. Other outcomes could include reformulation to reduce sugar content or changes in marketing to encourage purchasers to switch to low-sugar products or small portion sizes. Different responses will have different effects on consumption patterns for soft drinks and hence determine the health effects of the levy.^6^ The aim of this study is to appraise the health effect of various discrete industry responses so that legislation for the soft drinks levy can be designed to maximise health gain.

**Research in context**

**Evidence before this study**

The UK Government announced a soft drinks industry levy in March, 2016. Multiple observational and modelling studies have analysed the effect of soft drink taxes in other international settings; however, the UK would be first to introduce a tiered industry levy (high tax for drinks with >8 g of sugar per 100 mL, moderate tax for 5–8 g, and no tax for <5 g) rather than a sales tax, as has been applied elsewhere. To our knowledge, no analyses of its potential impact have been done and no international precedent exists from which to predict the potential response of soft drink manufacturers to the levy.

**Added value of this study**

This study, to our knowledge, is the first to estimate the health impact of the UK soft drinks industry levy. It focuses on obesity, diabetes, and oral health, for which evidence of a causal link between soft drink consumption and health is strongest. Previous evidence has suggested that soft drink taxes lead to price rises and subsequent reductions in purchases of targeted drinks. This study goes further and estimates the effects of six scenarios to illustrate the relative health impacts of three possible industry responses to the levy: reformulation, price rises, and changes to product market share.

**Implications of all the available evidence**

Each of the three responses modelled could lead to important health gains, with industry likely to react to the levy using a combination of all three. This study extends previous analyses of the effect of soft drink taxes to show the benefits of reformulation stimulated by the tiered levy. Our analyses show that substantial health benefits could occur if the levy stimulates reformulation. Further important health benefits from price changes will be mitigated if industry spread the price increase across their entire portfolio. Increases in market share for mid-sugar and low-sugar drinks could have substantial health benefits, but only if the market share comes at the expense of high-sugar drinks rather than people shifting from low-sugar to mid-sugar drinks.

## Methods

### Scenarios

We developed a comparative risk assessment model to estimate the effects of SSB reformulation, price changes, and changes to SSB market share on obesity, dental caries, and type 2 diabetes in the UK (figure). The baseline for the model was the 2014 UK population and we took all data for the model from the closest year for which data were freely available (appendix). We identified and modelled three possible industry responses. First, reformulation to reduce sugar concentration; second, a rise in price; and third, activities to change the relative market share of high-sugar, mid-sugar, and low-sugar drinks. For each of these responses, the magnitude of the response is uncertain. Informed by evidence where available and expert opinion, for each response we identified better-case and worse-case scenarios for reduction of sugar consumption, resulting in six scenarios (table 1).

**Figure:**
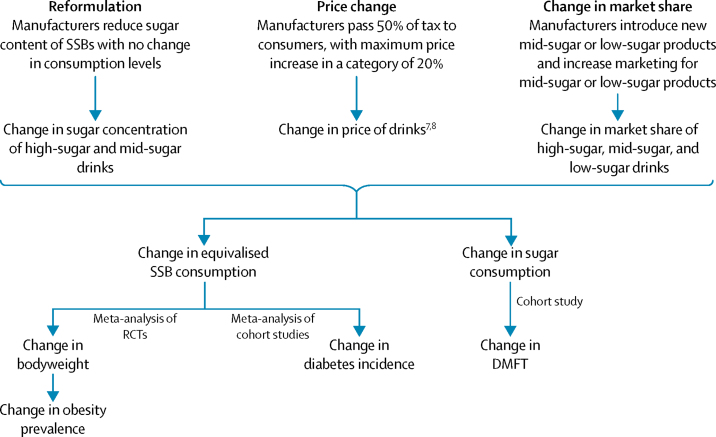
Conceptual model DMFT=decayed, missing, or filled teeth. RCT=randomised controlled trial. SSB=sugar-sweetened beverage.

**Table 1:** Simulated scenarios

	**Better case for sugar reduction**	**Worse case for sugar reduction**
Reformulation	Scenario 1*:* high-sugar drinks reduce sugar content by 30% and mid-sugar drinks by 15%	Scenario 2*:* mid-sugar and high-sugar drinks both reduce sugar content by 5%
Price change	Scenario 3: increase in price of high-sugar and mid-sugar drinks such that 50% of levy is passed on to consumers with a maximum 20% price rise	Scenario 4: increase in price of all packaged drinks* by the same percentage such that 50% of the tax is borne by customers
Change to SSB market share	Scenario 5: breakdown in sales of soft drinks shifts from 58% to 64% for low-sugar drinks, 6% to 12% for mid-sugar drinks, and 36% to 24% for high-sugar drinks	Scenario 6: breakdown in sales of soft drinks shifts to 55% for low-sugar drinks, 12% for mid-sugar drinks, and 33% for high-sugar drinks

Low-sugar drinks is less than 5 g of sugar per 100 mL, medium-sugar drinks is 5–8 g of sugar per 100 mL, and high-sugar drinks is more than 8 g of sugar per 100 mL. SSB=sugar-sweetened beverage.

* Including low-sugar or zero-sugar drinks, bottled water, fruit juice, and sweetened milk drinks, and not including tea, coffee, unsweetened milk, and alcohol.

We adopt the government definitions of high-sugar drinks as those with more than 8 g of sugar per 100 mL, mid-sugar drinks as those with 5–8 g of sugar per 100 mL, and low-sugar drinks as those with less than 5 g of sugar per 100 mL. Soft drinks are defined as all drinks with added sugar or sweetener; SSBs are drinks with added sugar, excluding milk-based drinks, tea, and coffee; concentrated SSBs are defined as SSBs that are intended to be diluted with water, and regular SSBs are intended to be drunk as sold.

Small producers will be excluded from the levy.^4^ We searched all soft drinks sold through the Tesco website and extracted the names of manufacturers. We used the Companies House website to identify manufacturers fulfilling the UK Government definition of a small company^9^ and identified 13 small companies, which together contributed 0·6% of total UK SSB sales. Therefore, we did not adjust our analyses to account for these companies.

The better-case reformulation scenario (scenario 1) assumed that industry would reduce the sugar concentration of high-sugar drinks by 30% and mid-sugar drinks by 15%. This assumption is based on the reformulation of Sprite and Lipton Ice Tea, which have both reduced their sugar concentration by 30% since 2013.^10^, ^11^ In the worse-case scenario (scenario 2), we assumed a 5% reduction in sugar concentration of both high-sugar and mid-sugar drinks. This assumption was based on Coca-Cola's pledge made to the Public Health Responsibility Deal of a 5% reduction in calories across their sparkling drink range between 2012 and 2014; they achieved a 5·3% reduction.^11^ Under both these scenarios, the volume consumed is assumed to remain constant.

To derive the price change scenarios, we used estimates from the Office for Budget Responsibility that the levy will be 18 pence per L on mid-sugar drinks and 24 pence per L on high-sugar drinks.^12^ Low-sugar drinks will not be taxed. Previous sugary drink taxes have been passed on at rates of between 50% and 100%,^13^, ^14^, ^15^ and if the tax was entirely passed through to consumers, high-sugar concentrated drinks would, on average, increase in price by 75% and high-sugar regular drinks would increase by 31% (table 2). Such price rises are markedly greater than in other examples of SSB taxes (most countries have adopted smaller tax rates and therefore, despite high pass-on rates, only result in a 5–15% price rise)^17^ and are larger than the 20% often cited as being necessary to affect substantial behavioural change and improve health.^18^

**Table 2:** Baseline price^16^ and change in price for the taxed drinks categories with different rates of tax pass-throughs and as modelled in scenario 3

	**Baseline price (pence per L)**	**Price with 100% pass-through (pence per L)**	**Price with 50% pass-through (pence per L)**	**Scenario 3 modelled price (pence per L)**
Concentrated high sugar	32·1	56·1 (+75%)	44·1 (+37%)	38·6 (+20%)
Concentrated mid sugar	40·1	58·1 (+45%)	49·1 (+22%)	48·1 (+20%)
Regular high sugar	77·6	101·6 (+31%)	89·6 (+15%)	89·6 (+15%)
Regular mid sugar	99·0	117·0 (+18%)	108·0 (+9%)	108·0 (+9%)

Percentages in parentheses indicate percentage change in baseline price.

We therefore assumed that 50% of the price increase would be passed on to purchasers and that companies would not increase prices by more than 20% (table 2). The better case for price change (scenario 3) assumed that the tax is passed on only through SSBs. However, major soft drink manufacturers produce various beverages. Therefore, in a worse case for price change (scenario 4), we assume that the levy is passed on evenly across all soft drinks (both diet beverages and SSBs), fruit juice, and bottled water, resulting in a 6% price rise. We modelled passing on 100% of the price increase to consumers as a sensitivity analysis. As stated by the UK Government, we applied tax rates to concentrated drinks given their price per litre as drunk, assuming a ratio of concentrate to water of one to four, as used by the British Soft Drinks Association (BSDA).^10^, ^19^ The appendix gives details of how we modelled the price change.

A change in SSB market shares might result from changes in product marketing, changing product size, or the introduction of new mid-sugar and low-sugar products. For example, the BSDA reports a 70% increase in expenditure on advertising of low-calorie or zero-calorie brands and growth in the sales of small pack sizes,^10^ and new mid-sugar products have emerged, such as Coca-Cola Life, which has 30% less sugar than does full-sugar Coca-Cola.^20^ Few data exist to inform the extent to which these activities drive changes in purchasing behaviour. However, the soft drinks industry has pledged to reduce energy intake from soft drinks by 20% from 2015 levels by 2020.^10^ To achieve this target, we calculate that the market share of high-sugar drinks would need to fall by 12% alongside a 6% increase for each of mid-sugar and low-sugar drinks, as shown in scenario 5, our better case for sugar reduction. The worse case (scenario 6) acknowledges that increased marketing of new mid-sugar drinks might lead consumers to switch to this category from low-sugar drinks. We assume that mid-sugar drinks double their market share alongside equal reductions in the market share of high-sugar and low-sugar drinks.

### Health impact modelling

We developed a comparative risk assessment model to estimate the effect of the changes to SSB purchasing on incidence of dental caries and type 2 diabetes and prevalence of obesity. Comparative risk assessment modelling requires identification of risk factor-disease pairs. In this case, the risk factor is SSB consumption and the diseases are dental caries, type 2 diabetes, and obesity. A two-step process then estimates the impact of the risk factor on the diseases. First, changes in the risk factor between baseline (current behaviour) and scenarios are estimated. Second, changes in the diseases as a result of changes in the risk factor are calculated using population impact fractions and applied to baseline levels of disease in the population. Such methods are common to the field of comparative risk assessment modelling^21^ and are based on model parameters representing baseline risk factor and disease status and the epidemiological relations between risk factors and diseases, which are assumed to be causal.

We sought parameters describing the direct relation between SSB consumption and health outcomes where possible from meta-analyses of randomised controlled trials where available or cohort studies (table 3).^22^, ^23^, ^24^, ^25^, ^26^, ^27^, ^28^ We modelled the relation between SSB consumption and diabetes and bodyweight as a function of SSB consumption. The reformulation scenarios (scenarios 1 and 2) assumed that SSB consumption stays constant, but the amount of sugar in the drinks reduces. To estimate the effect of these scenarios on obesity and diabetes, we derived estimates of equivalised SSB consumption, which rises and falls in direct proportion to volume of SSB consumed and average SSB sugar concentration. We standardised against the average sugar concentrations in drinks in the baseline scenario. For example, in the baseline scenario, the average sugar concentration of SSBs was 9·2 g per 100 mL and the average consumption was 213 mL per day. A reduction in the average sugar concentration to 8·2 g per 100 mL at the same level of consumption would have an equivalised SSB consumption of 190 mL per day. This equivalised SSB consumption arises because a reduction in consumption of SSBs from 213 mL per day to 190 mL per day at constant sugar concentrations would result in the same reduction of sugar as a reduction of sugar concentration from 9·2 g per 100 mL to 8·2 g per 100 mL at the same level of consumption. We used equivalised SSB consumption as an input for diabetes and obesity modelling in all scenarios.

**Table 3:** Model input parameters and data sources

	**Parameter**	**Data source**
Bodyweight	Increase in weight of 0·09 kg (95% CI −0·11 to 0·29) in adults and 0·45 kg (0·24–0·66) in children per additional 100 mL SSB consumed per day	Meta-analysis of randomised controlled trials of SSB consumption and bodyweight; two studies^22^, ^23^ identified and combined for adults and two^24^, ^25^ for children
Diabetes	Relative risk of incident diabetes of 1·42 (95% CI 1·19–1·69) per additional 250 mL serving per day for adults and children	Imamura et al^26^
Dental caries	Increase in number of decayed, missing, or filled teeth of 0·008 (95% CI 0·002–0·014) per person per year for every additional 10 g of sugar consumed per day	Bernabé et al^27^

SSB=sugar-sweetened beverage.

Uncertainty intervals reflect the uncertainty in baseline sugar drink sales and consumption, disease burden, sensitivity to price changes, and associations between sugar or sugary drink consumption and health outcomes. We estimated them using 5000 iterations of a Monte Carlo analysis, with model parameters drawn from the published or estimated uncertainty of each parameter (appendix).

We applied all results to the 2014 UK population^29^ and made separate estimates for each outcome by sex and age group using age-specific and sex-specific estimates of baseline SSB consumption and disease burden. Further details of the health impact model are in the appendix, including a sensitivity analysis in which the direct effect on weight of SSB consumption is replaced with an energy balance equation for comparison with other work that has used this method.^30^

### Role of the funding source

There was no specific funding source for this study. The corresponding author had full access to all the data and had final responsibility for the decision to submit for publication.

## Results

The better case for reformulation (scenario 1) resulted in a fall in mean sugar content of SSBs equivalent to a reduction of 58·5 mL (95% uncertainty interval [95% UI 54·5–62·6; 10 kcal [9–10]) of SSBs per person per day (table 4). This reduction is the largest among scenarios modelled. All simulated scenarios led to a fall in equivalised SSB consumption except for the worse case for market share, which resulted in a small increase. The largest falls for both sexes were among 11–18-year-olds who consume the largest volume of SSBs.

**Table 4:** Reduction in equivalised* volume of sugar-sweetened beverage consumed with each scenario

	**Reformulation**		**Price change**		**Change in market share**	
	Scenario 1	Scenario 2	Scenario 3	Scenario 4	Scenario 5	Scenario 6
**Male sex**						
Boys aged 4–10 years	61·7	11·2	34·5	12·4	38·6	−3·8
Boys aged 11–18 years	137·6	25·0	77·0	27·7	86·0	−8·6
Men aged 19–64 years	71·0	12·9	39·7	14·3	44·4	−4·4
Men aged ≥65 years	24·0	4·4	13·4	4·8	15·0	−1·5
**Female sex**						
Girls aged 4–10 years	51·9	9·5	29·1	10·4	32·5	−3·2
Girls aged 11–18 years	93·2	17·0	52·1	18·7	58·3	−5·8
Women aged 19–64 years	49·7	9·0	27·8	10·0	31·1	−3·1
Women aged ≥65 years	23·5	4·3	13·2	4·7	14·7	−1·5
**Total**						
Total (95% UI)	58·5 (54·5 to 62·6)	10·7 (10·0 to 11·4)	32·7 (30·3 to 35·3)	11·8 (10·9 to 12·7)	36·6 (34·9 to 38·3)	−3·6 (−3·8 to −3·4)

Data are in mL per person per day. UI=uncertainty interval.

* Where equivalisation results in the same sugar intake for each equivalised unit of sugar-sweetened beverage.

The reduction in obesity prevalence resulting from each scenario is estimated to be greatest after scenario 1 (better case for reformulation; table 5), leading to an estimated reduction of 144 383 (95% UI 5102–306 743) of 15 470 813 individuals with obesity, 0·9% of the obese population. This figure is compared with the better cases for price change (scenario 3), which reduces the obese population by 81 594 (3588–182 669; 0·5%), and with change in market share (scenario 5), which reduces the obese population by 91 042 (4289–204 903; 0·6%). Results varied by age, with larger reductions in the number of children with obesity than in that of adults in scenario 1. The relative reduction in obesity prevalence was predicted to be greater in male individuals than in female individuals because male individuals consume a greater volume of SSBs (appendix). Effect size estimates were significantly increased when an energy balance equation was used (appendix).

**Table 5:** Reduction in the number of obese individuals with each scenario

	**Reformulation**		**Price change**		**Change in market share**	
	Scenario 1	Scenario 2	Scenario 3	Scenario 4	Scenario 5	Scenario 6
**Male sex**						
Boys aged 4–10 years	29 227 (10·4%)	5524 (2·0%)	16 689 (6·0%)	6095 (2·2%)	18 592 (6·6%)	−1911 (−0·7%)
Boys aged 11–18 years	31 793 (6·0%)	5907 (1·1%)	17 987 (3·4%)	6521 (1·2%)	20 066 (3·8%)	−2033 (−0·4%)
Men aged 19–64 years	25 582 (0·5%)	4663 (0·1%)	14 324 (0·3%)	5149 (0·1%)	16 005 (0·3%)	−1596 (0·0%)
Men aged ≥65 years	3002 (0·2%)	547 (0·0%)	1680 (0·1%)	603 (0·0%)	1877 (0·1%)	−187 (0·0%)
**Female sex**						
Girls aged 4–10 years	16 455 (8·9%)	3097 (1·7%)	9374 (5·0%)	3418 (1·8%)	10 447 (5·6%)	−1070 (−0·6%)
Girls aged 11–18 years	17 581 (4·8%)	3257 (0·9%)	9930 (2·7%)	3595 (1·0%)	11 081 (3·0%)	−1120 (−0·3%)
Women aged 19–64 years	17 328 (0·3%)	3157 (0·1%)	9700 (0·2%)	3487 (0·1%)	10 839 (0·2%)	−1081 (0·0%)
Women aged ≥65 years	3415 (0·2%)	622 (0·0%)	1911 (0·1%)	697 (0·0%)	2135 (0·1%)	−213 (0·0%)
**Total**						
Total; 95% UI	144 383 (0·9%); 5102 to 30 6743	26 774 (0·2%); 1276 to 63 806	81 594 (0·5%); 3588 to 182 669	29 555 (0·2%); 1379 to 69 804	91 042 (0·6%); 4289 to 204 903	−9211 (−0·1%); −22 776 to −485

Data are n (%). UI=uncertainty interval.

Across the scenarios modelled, the pattern of results seen with obesity is repeated for type 2 diabetes. Scenario 1 (better case for reformulation) resulted in an estimated 19 094 (95% UI 6920–32 678; incidence reduction of 31·1 per 100 000 person-years) fewer new cases of diabetes per year and scenario 6 (worse case for change in market share) led to an increase of 1238 (455–2359; incidence increase of 2·0 per 100 000 person-years) cases per year (table 6). However, by contrast with the obesity results, adults aged older than 65 years saw the largest absolute reduction in diabetes incidence, reflecting the positive association between age and disease burden.

**Table 6:** Reduction in the number of cases of diabetes per year with each scenario

	**Reformulation**		**Price**		**Change in market share**	
	Scenario 1	Scenario 2	Scenario 3	Scenario 4	Scenario 5	Scenario 6
**Male sex**						
Boys aged 4–10 years	71 (2·6)	13 (0·5)	40 (1·5)	15 (0·5)	45 (1·6)	−5 (−0·2)
Boys aged 11–18 years	224 (7·5)	44 (1·5)	131 (4·3)	49 (1·6)	145 (4·8)	−15 (−0·5)
Men aged 19–64 years	8364 (43·5)	1585 (8·2)	4783 (24·9)	1749 (9·1)	5327 (27·6)	−549 (−2·8)
Men aged ≥65 years	2539 (49·4)	469 (9·1)	1431 (27·8)	517 (10·1)	1598 (31·1)	−160 (−3·1)
**Female sex**						
Girls aged 4–10 years	52 (2·0)	10 (0·4)	29 (1·1)	11 (0·4)	33 (1·2)	−3 (−0·1)
Girls aged 11–18 years	223 (7·8)	43 (1·5)	128 (4·5)	47 (1·7)	143 (4·9)	−15 (−0·5)
Women aged 19–64 years	5192 (26·7)	972 (5·0)	2950 (15·1)	1073 (5·5)	3289 (16·9)	−336 (−1·7)
Women aged ≥65 years	2429 (38·8)	448 (7·2)	1369 (21·8)	495 (7·9)	1528 (24·4)	−1549 (−2·5)
**Total**						
Total; 95% UI	19 094 (31·1); 6920 to 32 678	3584 (5·8); 1289 to 6466	10 861 (17·7); 3899 to 18 964	3955 (6·4); 1420 to 7085	1528 (19·7); 4414 to 21 785	−1238 (−2·0); −2359 to −455

Data in parentheses are reductions in incidence per 100 000 person-years. UI=uncertainty interval.

All scenarios except for scenario 6 led to a fall in the numbers of teeth affected with dental caries (measured by the number of decayed, missing, or filled teeth [DMFT]; table 7). The better case for reformulation (scenario 1) had the largest effect size, reducing the annual incidence of DMFT by 269 375 (95% UI 82 211–470 928; incidence reduction of 4·4 per 1000 person-years). As with results for obesity and diabetes, the better case for change in market share (scenario 5) and price change (scenario 3) scenarios had the next largest effects respectively. Those aged 11–18 years were expected to have the greatest relative benefit because of their higher baseline SSB consumption than those aged older or younger than this age group (appendix).

**Table 7:** Reduction in number of decayed, missing, or filled teeth per year with each scenario

	**Reformulation**		**Price**		**Change in market share**	
	Scenario 1	Scenario 2	Scenario 3	Scenario 4	Scenario 5	Scenario 6
**Male sex**						
Boys aged 4–10 years	12 735 (4·6)	2318 (0·8)	7022 (2·6)	3376 (1·2)	8096 (2·9)	−741 (−0·3)
Boys aged 11–18 years	31 040 (10·3)	5650 (1·9)	17 268 (5·7)	7015 (2·3)	19 967 (6·6)	−1922 (−0·6)
Men aged 19–64 years	102 477 (5·3)	18 654 (1·0)	56 909 (3·0)	23 776 (1·2)	65 792 (3·4)	−6282 (−0·3)
Men aged ≥65 years	9239 (1·8)	1682 (0·3)	5081 (1·0)	2554 (0·5)	5924 (1·2)	−563 (−0·1)
**Female sex**						
Girls aged 4–10 years	10 225 (3·9)	1861 (0·7)	5645 (2·2)	2640 (1·0)	6496 (2·5)	−593 (−0·2)
Girls aged 11–18 years	19 977 (7·0)	3637 (1·2)	11 099 (3·9)	4610 (1·6)	12 808 (4·5)	−1216 (−0·4)
Women aged 19–64 years	72 625 (3·7)	13 220 (0·7)	40 270 (2·1)	17 240 (0·9)	46 516 (2·4)	−4397 (−0·2)
Women aged ≥65 years	11 056 (1·8)	2013 (0·3)	6086 (1·0)	3029 (0·5)	7119 (1·1)	−688 (−0·1)
**Total**						
Total; 95% UI	269 375 (4·4); 82 211 to 470 928	49 036 (0·8); 14 929 to 85 630	149 378 (2·4); 45 231 to 262 013	64 240 (1·1); 19 643 to 112 371	172 718 (2·8); 47 919 to 294 499	−16 401 (−0·3); −28 037 to −4604

Data in parentheses are reductions in incidence per 1000 person-years. UI=uncertainty interval.

In our sensitivity analysis where 100% of the levy is passed on to consumers, equivalised SSB consumption would reduce by 71 mL (95% UI 66–77; 11 kcal [10–12]) per person per day. This reduction would lead to 174 818 (7536–367 647) fewer individuals with obesity, 23 046 (8419–39 965) fewer cases of diabetes per year, and 324 488 (89 073–553 840) fewer DMFT per year.

## Discussion

The proposed UK soft drinks industry levy has the potential to reduce obesity prevalence, diabetes incidence, and dental caries incidence. The effect on health and the ranking of scenarios is sensitive to the manner in which industry responds to the levy and the uncertainty in the modelling. Our estimates suggest that the greatest benefits will result from reformulation, with less but still positive health effects after price changes and changes to SSB market share to increase the proportion of low-sugar drinks sold, although in the worse-case scenario for change in market share, the health effect was actually negative. Children will have the greatest relative health benefit in terms of obesity and caries, with absolute reductions in diabetes incidence rates increasing with age.

The main strength of this study is the timely assessment of a planned government policy by simulating a set of discrete scenarios for how industry might respond to the levy to inform the detail of the legislation. Other strengths include modelling of multiple health outcomes, use of age-specific and sex-specific data, use of own-price and cross-price elasticities for high-sugar, mid-sugar, and low-sugar drinks, and use of equivalised SSB consumption to allow for changes in both sugar content and SSB volume.

Uncertainty intervals estimate the uncertainty arising from model parameters; however, the greatest uncertainty is how the soft drinks industry will respond to the levy. Given this uncertainty, our results should not be read as precise estimates of the impact of the levy, but instead should be used to compare the relative effects of different scenarios. Moreover, industry is likely to respond with a blended approach that combines elements of reformulation, price changes, and marketing. Although the results have wide and overlapping uncertainty intervals, much of the uncertainty is correlated between scenarios. In all of the iterations of our Monte Carlo analysis, the best-case reformulation scenario was associated with the best health outcomes, which suggests that the ranking of scenarios is robust. We have not estimated uncertainty in how much of the levy is passed on to consumers (although a 100% pass-on is modelled as a sensitivity analysis) and we did not use child-specific estimates of the effect of SSB consumption on diabetes and dental caries incidence because of an absence of data available. We have assumed that disease risk from SSBs is dependent on the quantity of sugar consumed (more sugar leads to higher risk). Although findings from studies^24^, ^25^ have shown benefits from a swap from SSBs to artificially sweetened beverages, we are not aware of any studies that have described the effect of a swap of SSBs with a high sugar content to SSBs with a low sugar content.

We have not modelled a temporal component. The effects on DMFT could occur soon after the change in SSB consumption, and the trials^22^, ^23^, ^24^, ^25^ used to parameterise the relation between SSB consumption and weight suggest that falls in obesity would be expected within 6 months for adults and 12 months for children. The effects on type 2 diabetes could take longer to be realised than for obesity (median follow-up of observational studies^26^ used for this parameter ranged between 3·4 years and 21·1 years). We have also not modelled results for different subgroups. Individuals from different socioeconomic backgrounds, ages, and baseline consumption levels could respond differently to each industry response simulated. Finally, we have not modelled the long-term health benefits of falls in obesity, the possible educational role the levy might have in highlighting that SSBs cause disease,^31^ and the health effect of use of the revenue to improve school sport and nutrition.

This study is, to our knowledge, the first to appraise the potential health effects of the UK soft drinks industry levy. The results of our study vary from those of a report by Oxford Economics,^32^ which calculated the impact of a price change associated with a 100% pass-on of the levy to targeted products only, with no reformulation or market share (most similar to our scenario 3). The authors estimated that the levy would result in a 5 kcal per-person per-day fall in energy intake. Our sensitivity analysis of a 100% pass-on rate would result in a reduction of 11 kcal per person per day (before adjustment for BSDA sales figures). Two principal explanations exist for the difference. First, we estimate the average price before tax of dilutables as 22 pence per L, whereas Oxford Economics estimate it as £1·76 per L. This discrepancy is likely to be due to Oxford Economics applying the tax before dilution. Second, Oxford Economics used estimates of how consumers respond to price changes of diet and non-diet SSBs taken from our 2013 study estimating the effect on obesity of a 20% UK SSB tax.^30^ In our present study, we have calculated estimates separately for high-sugar, mid-sugar, and low-sugar drinks.

Our 2013 study^30^ estimated that a 20% price rise would lead to a 1·3% fall in the number of adults with obesity in the UK, compared with our scenario 3 estimate of 0·5% (after an average price rise of 15%). Our 2013 study did not estimate price elasticities separately for high-sugar and mid-sugar drinks, did not quantify the effect of the tax on children, and did not adjust for BSDA sales figures. It also used an energy balance equation rather than quantifying the direct effect of SSBs on bodyweight, which estimates larger effects on bodyweight than does quantifying the direct effect of SSBs on bodyweight. Conversely, in this study, we used an estimate of the direct relation between bodyweight and SSBs, which might more accurately represent substitution and other compensatory mechanisms secondary to changes in sugar (and energy) consumed from SSBs than use of an energy balance equation. This analysis substantiates our 2013 findings of greater relative reductions in obesity among younger adults than among older adults. This finding is explained by teenagers and young adults drinking more SSBs than do old adults and trial data suggesting that SSBs have a greater effect on weight gain in children than in adults.^23^, ^24^, ^25^, ^26^

Considering reformulation, Ma and colleagues^33^ estimated that a 40% reduction in sugar across all SSBs in the UK would lead to 800 000 fewer individuals with obesity. This estimate is substantially higher than our estimate in scenario 1 (about 144 400), which assumed a 30% reduction in sugar content of high-sugar drinks and 15% reduction in that of mid-sugar drinks. This discrepancy is, in part, due to Ma and colleagues estimating that the average reduction in energy consumed would be approximately twice our estimate and then using an energy balance equation to estimate the effect of energy intake on weight. Of note, estimates for the reduction in diabetes were similar between our study and Ma and colleagues' study. We also recognise that uncertainties exist around all parameters in the model that will affect the comparison of results with those from other simulation studies.

The UK soft drinks industry levy has the potential to lead to important improvements in population health, particularly among children. Policy makers should engage with stakeholders to encourage responses to the levy that will maximise the potential health benefits of the new policy. Our results show the need for ongoing monitoring of the implementation strategies adopted by industry alongside modelling to estimate the long-term health consequences of their actions. Our results suggest that, of the scenarios modelled, reformulation would lead to the largest health benefits. Price rises and changes to product market share might also lead to important improvements in health. However, effects would be attenuated if manufacturers chose to pass the tax on to purchasers across all drinks or other products in their portfolio rather than just those targeted by the levy. Moreover, negative health effects might arise if the increase in market share of mid-sugar drinks comes at the expense of low-sugar drinks. Conversely, further health benefits might be realised if manufacturers pass on more than 50% of the levy to consumers or choose to reformulate to a greater extent than that modelled (as announced by Tesco^34^ and Lucozade Ribena Suntory^35^).

The UK soft drinks industry levy could have valuable population health benefits, but the magnitude of its health impact will depend on how industry responds. The detail of the levy is yet to be decided, but we show important health benefits that could be maximised by substantial product reformulation, with further health gains arising through raising the price of high-sugar and mid-sugar drinks and increasing the market share of low-sugar products.
