# Supplementary material for: Health impact assessment of the UK soft drinks industry levy: a comparative risk assessment modelling study
Source: Lancet Public Health. 2016 Dec 16;2(1):e15–22. doi: 10.1016/S2468-2667(16)30037-8 (PMC5543265; doi:10.1016/S2468-2667(16)30037-8)

THE LANCET Public Health

Supplementary appendix

This appendix formed part of the original submission and has been peer reviewed. We post it as supplied by the authors.

Supplement to: Briggs ADM, Mytton OT, Kehlbacher A, et al. Health impact assessment of the UK soft drinks industry levy: a comparative risk assessment modelling study. *Lancet Public Health* 2016; published online Dec 15. http://dx.doi.org/10.1016/S2468-2667(16)30037-8.

A health impact assessment of the UK soft drinks industry levy: a comparative risk assessment modelling study

Adam D M Briggs, Oliver T Mytton, Ariane Kehlbacher, Richard Tiffin, Ahmed Elhussein, Mike Rayner, Susan A Jebb, Tony Blakely, Peter Scarborough

Supplementary material: Description of the modelling process

Modelling price change

The effect of a price change on purchases of SSBs and on complementing and substituting drinks was modelled using own- and cross-price elasticities. Own-price elasticities describe how purchases of a good change due to a price increase, and cross-price elasticities describe the effect of the price change on other goods. For example, if bread has an own price elasticity of -1.0 and a cross price elasticity with butter of -0.2 and of 0.1 with cereal, a 10% increase in the price of bread will result in 10% less bread, 2% less butter (butter is a complement) and 1% more cereal (cereal is a substitute) being purchased.

Conditional own- and cross-price elasticities were estimated for 25 food and drink categories; supplementary figure 1 shows these categories and illustrates the demand system hierarchy. Demand systems 1 to 5 were estimated using 2010 Living Costs and Food Survey (LCF) data and using an Almost Ideal Demand System (AIDS)[1,2] with infrequency of purchase[3] which assumes that censoring arises because households consume from stock. Adding up, symmetry, homogeneity and concavity are imposed in the estimation. Because the covariance matrix is singular we drop one of the share equations, obtaining estimates that are invariant to which equation is dropped.[4] Food categories are aggregated using the EKS quantity index[5,6] which is a multi-lateral version of the superlative Fisher Ideal index, and is used to compute the implicit price index. A superlative index offers some mitigation towards the concerns over the potential endogeneity of prices.[7] The LCF data are a cross-section of 5,236 households which are chosen using multi-stage stratified random sampling. Data on 270 categories of food expenditures and quantities purchased for consumption at home are collected from each household over a two week period using food diaries and these periods are distributed across the year for different households. For the estimation of the elasticities of concentrated and regular mid- and high- sugar drinks (demand systems 6 and 7), we used selected product codes from the 2010 Kantar World Panel UK data, a panel of 30,000 households demographically representative of Great Britain. These data are collected over 52 weeks and we therefore used an AIDS with double hurdle model that assumes censoring arises because households prefer to not purchase a given drink. Estimation was carried out using Markov chain Monte Carlo methods where the Gibbs sampler draws 10,000 samples sequentially from the conditional posterior distributions after a burn-in of 2,000. The conditional elasticities of the seven demand systems were combined to obtain unconditional elasticities which allow for expenditure to vary within all food and drinks categories.[8] The own and cross-price elasticity estimates was used to estimate the change in consumption of each drink category by multiplying the elasticity matrix with the price changes modelled. The percentage change in price is estimated by applying the proposed levy rates to expenditure of each taxed drink category in LCF, 2014. See supplementary table 1 for price elasticity matrix.

We assumed that the price elasticities apply uniformly across the population whereas in reality individuals may respond differently to the price rise based on their age, baseline consumption, and socio-economic group.[9,10] Furthermore, price elasticities are estimated using data containing price variations which are smaller than the price increases modelled. Therefore greater price increases have greater uncertainty surrounding the estimated responses of households.

Modelling health outcomes

We estimate the effect of changing SSB and sugar consumption on obesity prevalence in the UK, and on annual diabetes incidence and incidence of decayed, missing, and filled teeth (DMFT).

Baseline drink consumption was taken from LCF, 2014.[11] Drinks were categorised into milk, water, fruit juice, soft drinks regular low calorie, soft drinks regular not low calorie, soft drinks concentrated low calorie, soft drinks concentrated not low calorie, tea and coffee, other beverages, beer, wine, and other alcohol. Regular soft drinks include carbonates, energy drinks, and fruit juice with added sugar.

The LCF divides SSBs into *soft drinks concentrated, not low calorie,* and *soft drinks regular, not low calorie.* In order to estimate the effect of the levy on mid-sugar drinks, these categories were split into high-sugar and mid-

sugar drink categories using the following method. The volume purchased and sugar concentration of each of the *not low-calorie soft drink* categories is reported by the LCF. The volume purchased for each of the mid-sugar categories was estimated by assuming that the concentration of sugar in drinks in the high-sugar category is 10g sugar/100ml and in the mid-sugar category is 6.5g sugar/100ml, which is supported by an analysis of Kantar World Panel Data.[12] Using these data, the ratio of high- to mid-sugar drink volume purchased for each of regular and concentrated drinks was calculated and applied to the volume of *not low-calorie soft drinks* purchased thereby estimating the baseline volume of each of mid-sugar drinks and high-sugar drinks.

The volume purchased within some drinks categories in the LCF is considerably lower than industry reported sales data from the British Soft Drinks Association (BSDA).[13] Therefore, as with previous studies,[14] purchases of fruit juice, water, regular soft drinks and concentrated soft drinks were initially adjusted upwards based on 2015 industry reported total volume sales.[13] Volume consumed was assumed to be the same as that purchased minus wastage estimated for carbonated soft drinks, bottled water, fruit juice, and concentrated soft drinks by the Waste and Resources Action Programme (WRAP), a survey of approximately 1,800 households in the UK with detailed measurement of weight and types of and drink waste.[15]

As the LCF reports at the household level, age and sex specific estimates of drinks consumed were derived by dividing the total volume of SSBs purchased per person in the LCF by the relative consumption of each drink category by age and sex reported by The National Diet and Nutrition Survey rolling programme, 2008/09-2011/12 (NDNS).[16] The NDNS is a four day dairy of food consumption for both children and adults and the 2008/09 – 2011/12 data are based on data collected for over 6,000 participants.

We have assumed there is no excess health risk associated with artificially sweetened beverages (ASBs). While we note that some studies have reported that ASBs are associated with obesity and type 2 diabetes in prospective studies many authors suggest these associations may be due to reverse causation and there is no strong plausible biological explanation for an aetiological association.[17,18] However we note that ASBs are linked to enamel erosion, and the harm associated with this has not been modelled.[19]

Data from BSDA 2015 suggest that the market share of low- middle-, and high-sugar drinks is 58%, 6%, and 36% respectively, compared to our estimates from LCF 2014 of 50%, 17% and 33%. Therefore for scenarios 5 and 6, baseline volumes of concentrated and non-concentrated soft drinks consumed were adjusted to fit BSDA reported 2015 market shares. The new market shares described in table 1 of the manuscript were then compared to this new baseline. The concentration of sugar in each category and their prices were assumed to remain the same.

Finally, the model is static meaning that we do not estimate the effect of scenarios on disease over time and have not incorporated current disease or risk factor trends. Results therefore relate to 2014 disease incidence and prevalence rates.

Modelling obesity

The relationship between SSB consumption and body weight was derived following a random effects meta-regression of randomised controlled trials describing the change in body weight following change in volume of SSBs consumed. No published meta-regressions exist and therefore a search of the literature was completed in Medline and four trials were identified and combined, two in children[20,21] and two in adults.[22,23] Based on these data, we assumed a linear relationship between body weight and SSB consumption; for every additional 100ml of SSB consumed per day, we assumed 0.09kg (-0.11 to 0.29) of additional weight in adults and 0.45kg (0.24 to 0.66) in children. Weight increases were then converted into change in body mass index (BMI) using age and sex specific measures of height and weight from the Health Survey for England (HSfE, assumed to apply to the UK population).[24] Overweight and obesity in children was defined as being above the 91st and 98th centile respectively for weight based on sex specific Royal College of Paediatrics and Child Health growth charts.[25] For both of the childhood studies included in the meta-analysis, body weight was recorded one year after the introduction of the intervention. Overweight and obesity in adults was defined as a body mass index (BMI) of 25-30kg/m^2 and >30kg/m^2 respectively. For the adult studies included in the meta-analyses, body weight was recorded six months after introduction of the intervention.

We assumed that the effect of changing SSB consumption on diabetes and dental caries would be the same in adults and children whereas we assumed different effect sizes among adults when modelling obesity. The differences in effect size reported by trials estimating the effect of SSB consumption on weight gain may in part be due to the adult trials reporting after six months whereas the trials in children reported at 1 year post intervention.[20–23] The adult trials used may under-report the potential total weight loss, meaning our obesity

results are conservative. Results estimate the number of obese individuals in 2014 had the population been consuming SSBs as modelled in each scenario rather than current consumption. We repeated the obesity estimates using Christiansen and Garby's energy balance equation, which estimates significantly greater falls in obesity prevalence (as used by Briggs et al., see supplementary table 3).[26,27] Hall and Jordan's dynamic model estimates a two-fold greater loss of weight than we estimate after six months (but with further weight loss to be expected if the calorie change is maintained), again suggesting that we may be conservative in our estimates.[28]

Modelling diabetes
We assumed a relative risk of 1.42 (95% confidence interval: 1.19 to 1.69) per 250ml SSB serving for diabetes incidence, based on the results of a random effects meta-analysis of 17 prospective cohort studies with median follow up ranging from 3.4 years to 21.1 years.[18] This estimate was unadjusted for obesity as obesity is on the causal pathway between SSB consumption and type 2 diabetes. We assumed no relationship between artificially sweetened beverages and type 2 diabetes. It was assumed that the same relative risk applies equally to both adults and children. Baseline incidence of diabetes by age and sex was taken from Holden et al.[29]

Modelling dental caries
We were unable to identify data to estimate the dose-response of SSB consumption on dental caries. We therefore assumed that for every additional 10g sugar consumed per day there would be 0.008 (0.002 to 0.014) extra DMFT per person in a given year. This estimate was from an 11 year-long longitudinal study of sugar consumption and oral health in Finnish adults controlling for a variety of confounders including socio-economic status and tooth brushing habits, the time for DMFT to manifest from a change SSB consumption was not observed.[30] Change in sugar consumption was estimated across all drinks consumed and not only change in SSB consumption. We assumed that there was no substitution from drinks to food when estimating the change in sugar consumption.

Uncertainty analysis
We conducted a Monte Carlo analysis with 5000 iterations to quantify the uncertainty in the modelled results due to uncertainty around the model parameters. In each iteration, the model parameters were drawn randomly from a specified distribution and the six scenarios were modelled using this random set of model parameters. Results for the iteration were saved, and uncertainty intervals for the results were based on the 2.5th and 97.5th percentiles of the results across the 5000 iterations. Uncertainty around the following parameters were included in the analysis: baseline consumption of drinks categories estimated by the LCF; volume of sales of drinks categories estimated by the BSDA; percentage of waste of food categories estimated by WRAP; sugar drink consumption by age and sex estimated by the NDNS; diabetes incidence by age and sex estimated by Holden et al.;[29] height and weight by age and sex estimated by the HSfE; own price elasticities for the soft drinks categories; and the parameters associating changes in sugar or sugar drink consumption and DMFT, diabetes incidence (drawn from the literature), and obesity prevalence (bespoke meta-analyses of two randomised controlled trials for both children[20,21] and adults).[22,23] Due to heterogeneity of study design we conducted random effects meta-analyses, therefore the parameters are averages of results reported in the literature, weighted by the inverse of the standard error. For most of these parameters, the prior distributions were estimated using data from the source material. This was not possible for some of the parameters, where there are no published estimates of their uncertainty. In these cases, we categorised our confidence in the data source as either 'confident' or 'moderately confident'. For data sources categorised as 'confident' (data from LCF, BSDA, Holden et al.) we used normal distributions with the published estimate as the mean and set the standard deviation as 5% of the mean. For those categorised as 'moderately confident' (data from WRAP), we set the standard deviation as 10% of the mean.

Due to unknown covariance between own-price and cross-price elasticities, we were unable to conduct a full probabilistic uncertainty analysis of the uncertainty due to the price elasticity matrix. The analysis conducted here is likely to capture most of this uncertainty (since the own-price elasticities drive most of the price effects from the price elasticity matrix) but some uncertainty associated with price change will be underestimated.

References

1 Deaton AS, Muellbauer J, Deaton A, Muellbauer J. An Almost Ideal Demand System. *Am Econ Rev* 1980; **70**: 312–26.
2 Department for the Environment Food and Rural Affairs. Family Food 2010. London, 2011.
3 Tiffin R, Arnoult M. The demand for a healthy diet: estimating the almost ideal demand system with infrequency of purchase. *Eur Rev Agric Econ* 2010; **37**: 501–21.
4 Barten AP. Maximum likelihood estimation of a complete system of demand equations. *Eur Econ Rev* 1969; **1**: 7–73.

5 Elteto O, Koves P. On a problem of index number computation relating to international comparison. *Statisztikai Szle* 1964; **42**: 507–18.

6 Szulc B. Indices for multiregional comparisons. *Prz Stat* 1964; **3**: 239–54.

7 Diewert WE. Exact and superlative index numbers. *J Econom* 1976; **4**: 115–45.

8 Edgerton DL. Weak Separability and the Estimation of Elasticities in Multistage Demand Systems. *Am J Agric Econ* 1997; **79**: 62–79.

9 Tiffin R, Kehlbacher A, Salois M. The Effects of A Soft Drink Tax in the UK. *Health Econ* 2015; **24**: 583–600.

10 Finkelstein EA, Zhen C, Nonnemaker J, Todd JE. Impact of targeted beverage taxes on higher- and lower-income households. *Arch Intern Med* 2010; **170**: 2028–34.

11 Office for National Statistics. Family Spending: 2015. A report on the Living Costs and Food Survey 2014. London, 2015.

12 Oxford Economics. The economic impact of the soft drinks levy. Oxford, 2016 http://www.britishsoftdrinks.com/write/MediaUploads/Publications/The_Economic_Impact_of_the_Soft_Drinks_Levy.pdf.

13 British Soft Drinks Association. Leading the Way. Annual report 2016. London, 2016.

14 Ma Y, He FJ, Yin Y, Hashem KM, MacGregor GA. Gradual reduction of sugar in soft drinks without substitution as a strategy to reduce overweight, obesity, and type 2 diabetes: a modelling study. *Lancet Diabetes Endocrinol* 2016; **4**: 105–14.

15 Quested T, Ingle R, Parry A. Household Food and Drink Waste in the United Kingdom 2012. Banbury, 2013.

16 Public Health England. National Diet and Nutrition Survey: Results from Years 1 - 4 (combined) of the Rolling Programme (2008/2009 – 2011/12). London, 2014.

17 Pereira MA. Sugar-sweetened and artificially-sweetened beverages in relation to obesity risk. *Adv Nutr* 2014; **5**: 797–808.

18 Imamura F, O'Connor L, Ye Z, *et al.* Consumption of sugar sweetened beverages, artificially sweetened beverages, and fruit juice and incidence of type 2 diabetes: systematic review, meta-analysis, and estimation of population attributable fraction. *BMJ* 2015; **351**: h3576.

19 Nadimi H, Wesamaa H, Janket S-J, Bollu P, Meurman JH. Are sugar-free confections really beneficial for dental health? *Br Dent J* 2011; **211**: E15.

20 de Ruyter JC, Olthof MR, Seidell JC, Katan MB. A trial of sugar-free or sugar-sweetened beverages and body weight in children. *N Engl J Med* 2012; **367**: 1397–406.

21 Ebbeling CB, Feldman HA, Chomitz VR, *et al.* A randomized trial of sugar-sweetened beverages and adolescent body weight. *N Engl J Med* 2012; **367**: 1407–16.

22 Maersk M, Belza A, Stødkilde-Jørgensen H, *et al.* Sucrose-sweetened beverages increase fat storage in the liver, muscle, and visceral fat depot: a 6-mo randomized intervention study. *Am J Clin Nutr* 2012; **95**: 283–9.

23 Tate DF, Turner-McGrievy G, Lyons E, *et al.* Replacing caloric beverages with water or diet beverages for weight loss in adults: main results of the Choose Healthy Options Consciously Everyday (CHOICE) randomized clinical trial. *Am J Clin Nutr* 2012; **95**: 555–63.

24 Health and Social Care Information Centre. Health Survey for England 2014. London, 2015.

25 Royal College of Paediatrics and Child Health. School age charts and resources. 2015. http://www.rcpch.ac.uk/improving-child-health/public-health/uk-who-growth-charts/school-age-2-18-years/school-age-charts-an.

26 Christiansen E, Garby L. Prediction of body weight changes caused by changes in energy balance. *Eur J Clin Invest* 2002; **32**: 826–30.

27 Briggs ADM, Mytton OT, Kehlbacher A, Tiffin R, Rayner M, Scarborough P. Overall and income specific effect on prevalence of overweight and obesity of 20% sugar sweetened drink tax in UK: econometric and comparative risk assessment modelling study. *BMJ* 2013; **347**: f6189.

28 Hall KD, Jordan PN. Modeling weight-loss maintenance to help prevent body weight regain. *Am J Clin Nutr* 2008; **88**: 1495–503.

29 Holden SH, Barnett AH, Peters JR, *et al.* The incidence of type 2 diabetes in the United Kingdom from 1991 to 2010. *Diabetes Obes Metab* 2013; **15**: 844–52.

30 Bernabé E, Vehkalahti MM, Sheiham A, Lundqvist A, Suominen AL. The Shape of the Dose-Response Relationship between Sugars and Caries in Adults. *J Dent Res* 2016; **95**: 167–72.

Supplementary table 1. Price elasticity matrix

	Dairy & Eggs	Meat & Fish	Fats & Starches	Fruits & Nuts	Veg	Milk	Water	Fruit Juice	SD conc, diet	*SD conc, regular*	*SD conc, mid-range*	SD diet	*SD regular*	*SD mid-range*	Tea & Coffee	Other beverages	Beer	Wine	Other alcohol
Dairy & Eggs	-0.534	0.000	-0.004	0.000	0.000	-0.001	0.000	0.000	0.000	0.000	0.000	0.000	-0.003	0.000	-0.003	0.000	0.000	0.002	0.000
Meat & Fish	0.000	-0.633	-0.005	0.000	0.000	-0.001	0.000	0.000	0.000	0.000	0.000	0.000	-0.003	0.000	-0.003	0.000	-0.002	-0.015	-0.001
Fats & Starches	0.000	-0.003	-0.564	0.000	0.000	-0.005	0.000	0.000	0.000	-0.002	-0.002	-0.002	-0.016	-0.002	-0.016	0.000	0.001	0.010	0.001
Fruits & Nuts	0.000	0.000	0.001	-0.809	0.000	0.002	0.000	0.000	0.000	0.001	0.001	0.001	0.006	0.001	0.006	0.000	-0.003	-0.021	-0.002
Veg	0.000	-0.002	-0.004	0.000	-0.503	-0.003	0.000	0.000	0.000	-0.001	-0.001	-0.001	-0.008	-0.001	-0.008	0.000	-0.001	-0.004	0.000
Milk	0.000	0.000	-0.005	0.000	0.000	-0.971	0.000	0.000	0.000	0.003	0.002	0.002	0.018	0.002	0.017	0.000	0.002	0.017	0.002
Water	0.000	0.000	-0.001	0.000	0.000	0.017	-0.835	0.000	0.000	0.001	0.001	0.001	0.010	0.001	-0.041	0.000	0.001	0.005	0.000
Fruit Juice	0.000	0.000	-0.004	0.000	0.000	0.014	0.000	-0.977	0.000	0.004	0.002	0.004	0.026	0.004	0.024	0.000	0.002	0.013	0.001
SD conc, diet	0.000	0.000	-0.003	0.000	0.000	0.000	0.000	0.000	-0.940	0.103	0.068	0.010	-0.008	-0.001	0.007	0.000	0.001	0.009	0.001
SD conc, regular	0.000	0.000	-0.004	0.000	0.000	0.001	0.000	0.000	0.004	-0.881	-0.023	0.017	0.045	0.006	0.010	0.000	0.002	0.014	0.001
SD conc, mid-range	0.000	0.000	-0.004	0.000	0.000	0.001	0.000	0.000	0.005	-0.049	-0.879	0.018	0.046	0.007	0.011	0.000	0.002	0.015	0.001
SD diet	0.000	0.000	-0.006	0.000	0.000	0.001	0.000	0.000	0.000	-0.003	-0.002	-1.013	0.016	0.002	0.016	0.000	0.003	0.022	0.002
SD regular	0.000	0.000	-0.006	0.000	0.000	0.001	0.000	0.000	-0.001	-0.013	-0.009	0.003	-0.952	0.004	0.015	0.000	0.003	0.020	0.002
SD mid-range	0.000	0.000	-0.006	0.000	0.000	0.001	0.000	0.000	-0.001	-0.013	-0.009	0.003	0.035	-0.976	0.015	0.000	0.003	0.020	0.002
Tea & Coffee	0.000	0.000	-0.005	0.000	0.000	0.010	0.000	0.000	0.000	0.006	0.004	0.006	0.044	0.006	-0.938	-0.002	0.002	0.017	0.002
Other beverages	0.000	0.000	-0.001	0.000	0.000	0.002	0.000	0.000	0.000	0.002	0.001	0.001	0.011	0.001	0.099	-0.680	0.001	0.004	0.000
Beer	0.000	-0.002	0.003	0.000	0.000	0.005	0.000	0.000	0.000	0.002	0.001	0.002	0.015	0.002	0.015	0.000	-1.009	0.015	0.002
Wine	0.000	-0.003	0.005	0.000	0.000	0.007	0.000	0.000	0.000	0.003	0.002	0.003	0.022	0.003	0.021	0.000	0.000	-1.008	-0.001
Other alcohol	0.000	-0.002	0.003	0.000	0.000	0.005	0.000	0.000	0.000	0.002	0.001	0.002	0.015	0.002	0.015	0.000	0.003	0.000	-0.934

SD, soft drink; conc, concentrated

Supplementary table 2. Baseline SSB consumption from NDNS years 1-4

Age and sex group	Sugar drink consumption (ml/d)
Boys, 4-10	139
Boys, 11-18	310
Men, 19-64	160
Men, 65+	54
Girls, 4-10	117
Girls, 11-18	210
Women, 19-64	112
Women, 65+	53

SSB, sugar sweetened beverage; NDNS, National Diet and Nutrition Survey

Supplementary table 3. Change in obesity prevalence using an energy balance equation

Scenario	Change in number of obese individuals
1: Reformulation better case	908773 (502697 to 1203852)
2: Reformulation worse case	168549 (96762 to 229847)
3: Price change better case	484621 (272800 to 651200)
4: Price change worse case	206642 (112800 to 282900)
5: Market share better case	592034 (324255 to 791264)
6: Market share worse case	-59925 (-33520 to -83278)

Supplementary figure 1. Demand system hierarchy for economic modelling

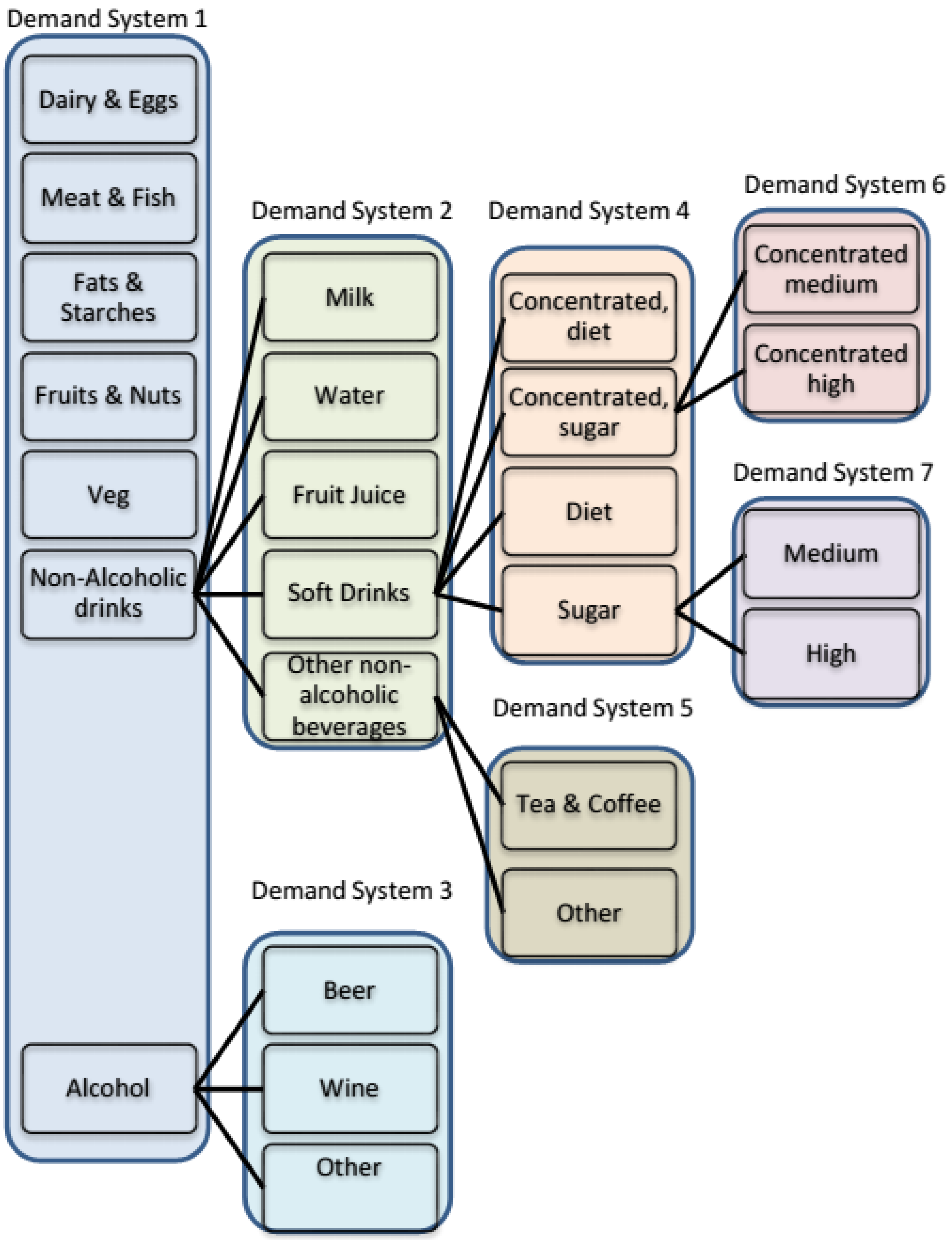

Supplement: Supplementary appendix [file mmc1.pdf]
